# Profiles of Beliefs about Alzheimer’s Disease Risk Factors and Prevention Strategies among Americans Aged 50+

**DOI:** 10.1093/geroni/igaf122.2530

**Published:** 2025-12-31

**Authors:** Elyse Couch, Fangli Geng, Eric Jutkowitz, Emmanuelle Belanger

**Affiliations:** Brown University, Providence, Rhode Island, United States; Brown University, Providence, Rhode Island, United States; University of Minnesota, Minneapolis, Minnesota, United States; Brown University, Providence, Rhode Island, United States

## Abstract

Increasing knowledge about the causes of dementia is essential for motivating engagement in healthy behaviors that can lower one’s risk of dementia. This study aimed to identify distinct patterns of beliefs regarding the importance of Alzheimer’s disease (AD) risk factors and the effectiveness of prevention strategies among Americans aged 50+. We analyzed data from 1,819 adults who participated in a 2010 Health and Retirement Study experimental module on AD knowledge and beliefs. On a 3-point Likert scale, participants rated the importance of AD risk factors (stress and genetics) for causing AD (1 = not important; 3 = very important) and the effectiveness of prevention strategies (physical activity, mental activity, diet, vitamins)(1 = not effective; 3 = very effective). We performed an unweighted latent profile analysis to identify three distinct profiles of beliefs about AD risk factors and prevention strategies and multinomial regression to identify demographic predictors of profile membership. Profile 1 (63% of participants) were uncertain about the importance of risk factors and the effectiveness of prevention strategies; Profile 2 (15% of participants) strongly believed in the effectiveness of all prevention strategies, and Profile 3 (21% of participants) rated genetics as the most important risk factor but did not believe in the effectiveness of prevention strategies. Demographic characteristics, such as education and race, were associated with profile membership. Public health messaging about AD prevention should be tailored to the beliefs of different demographic groups.

